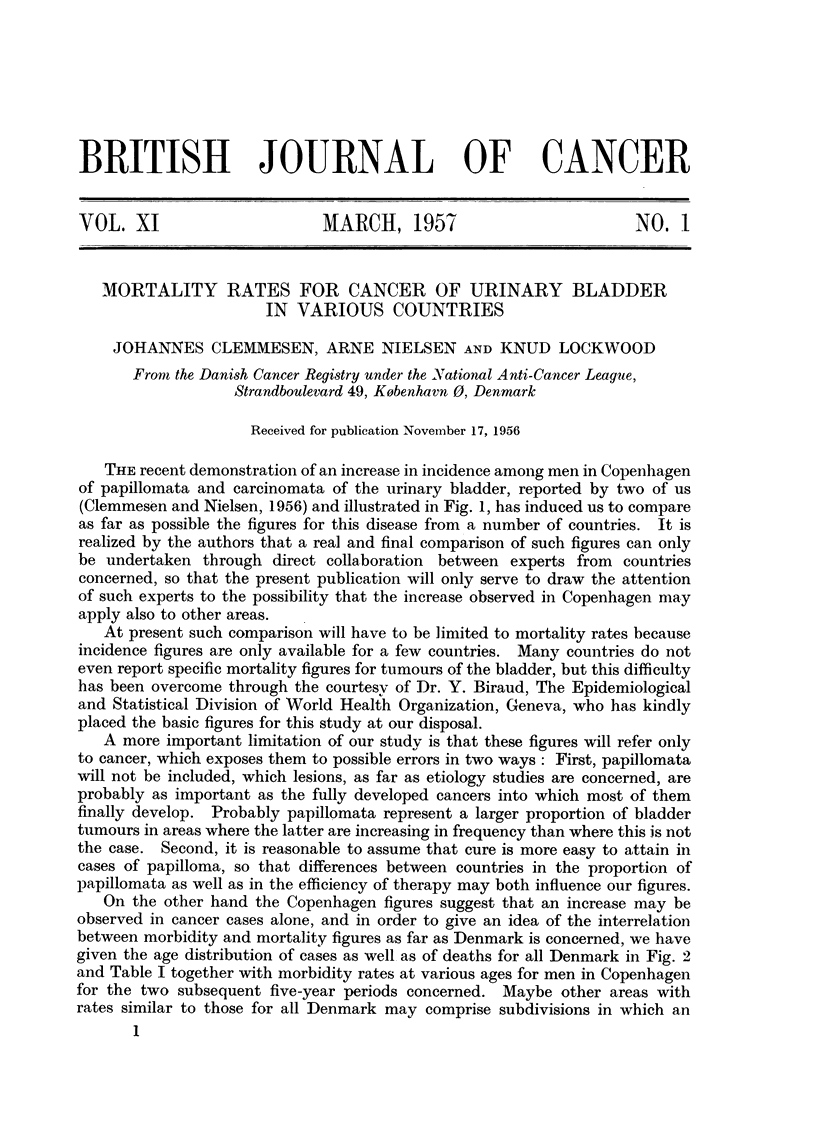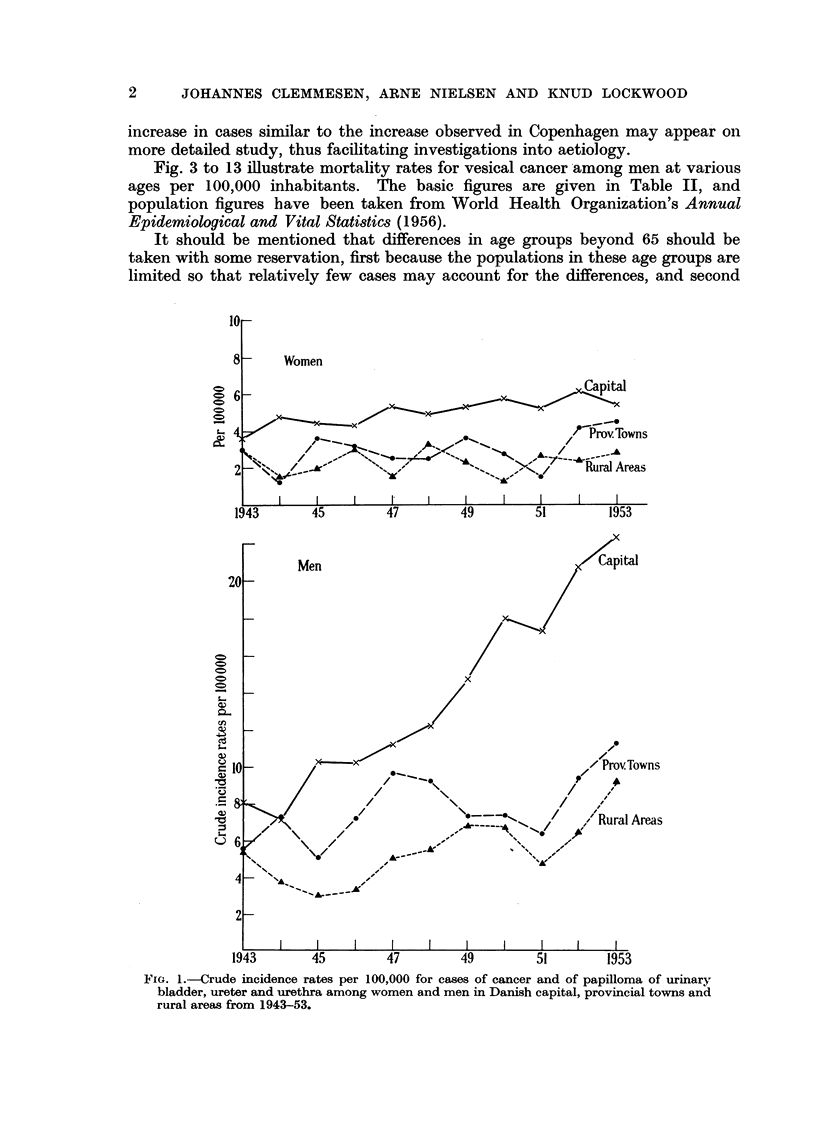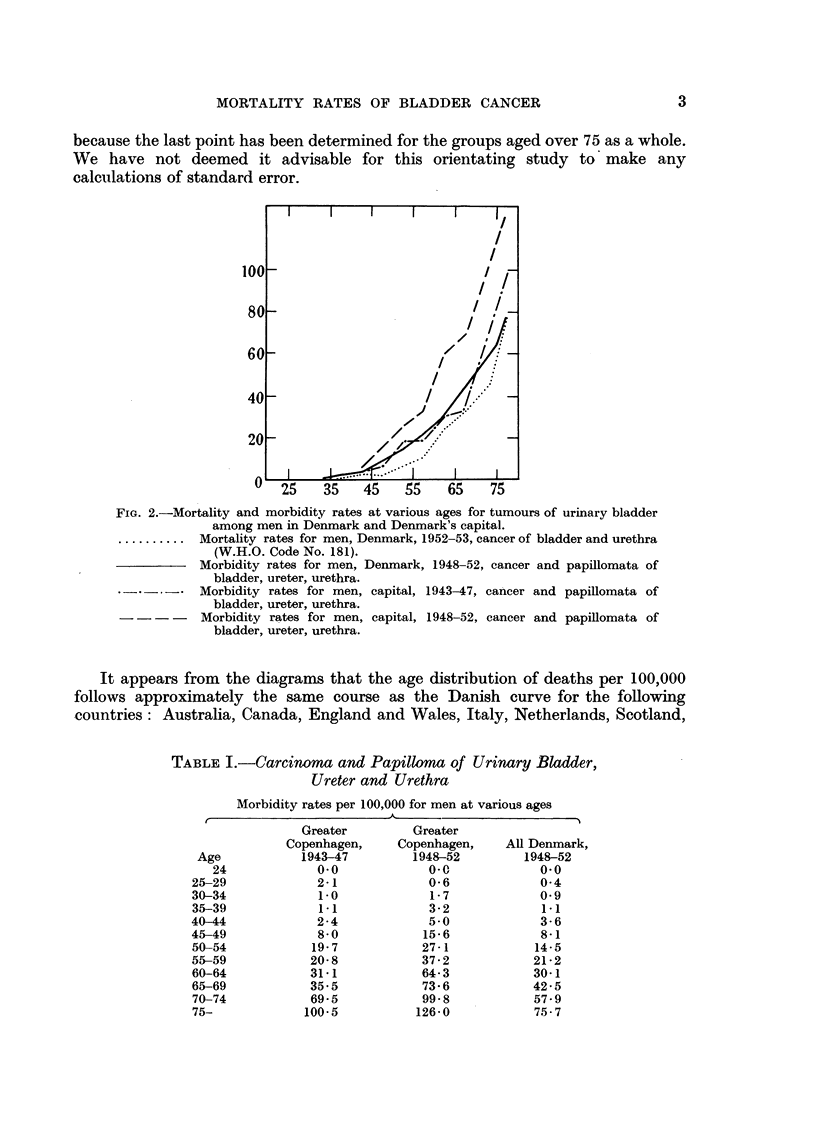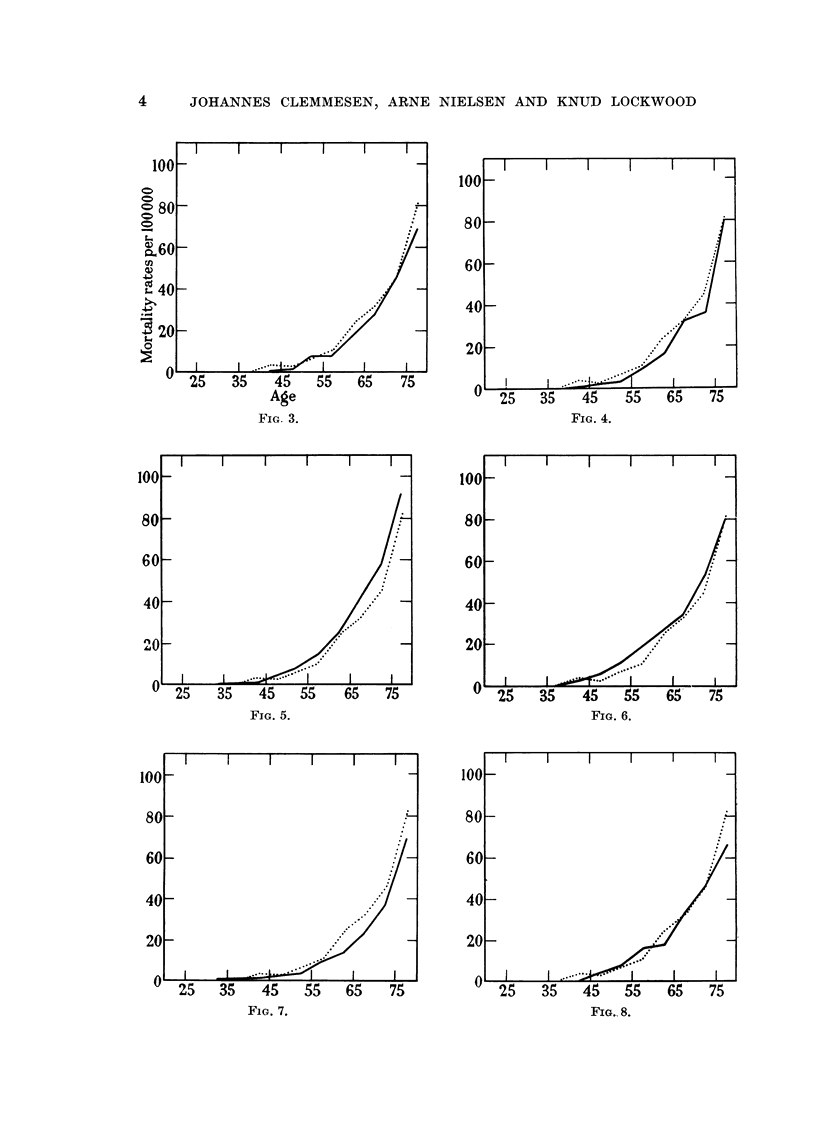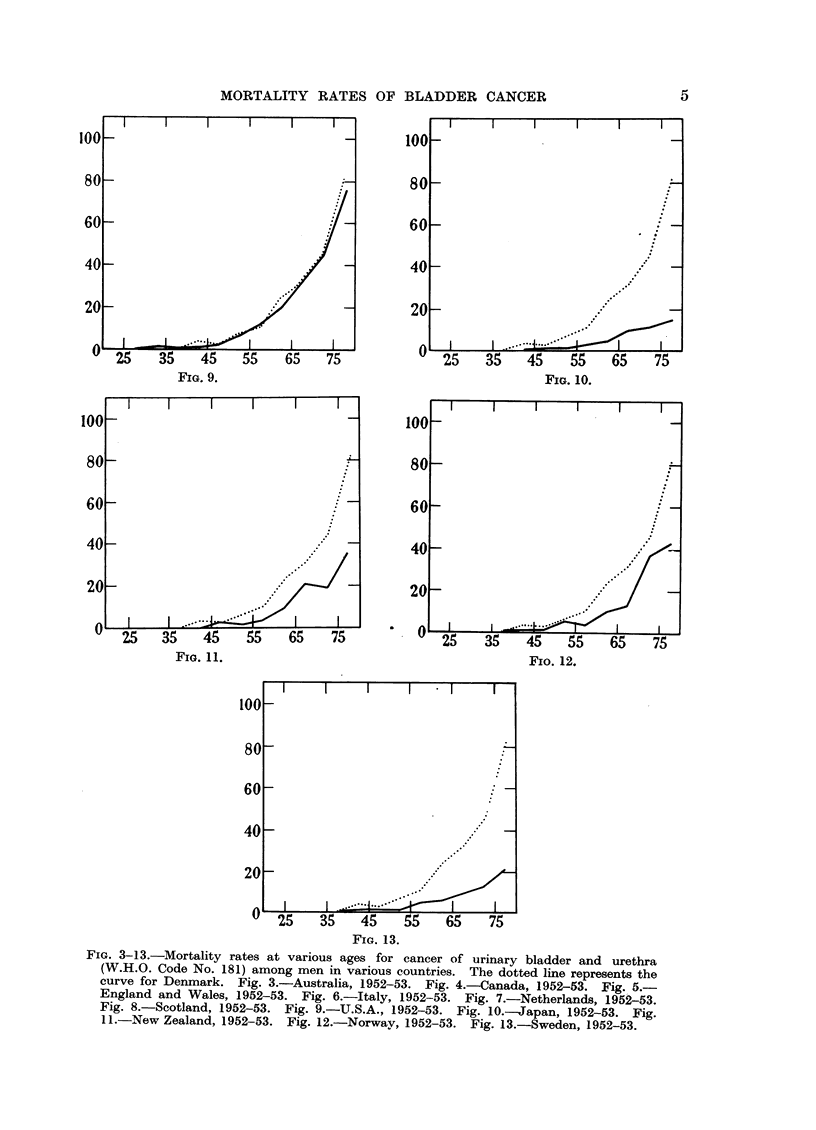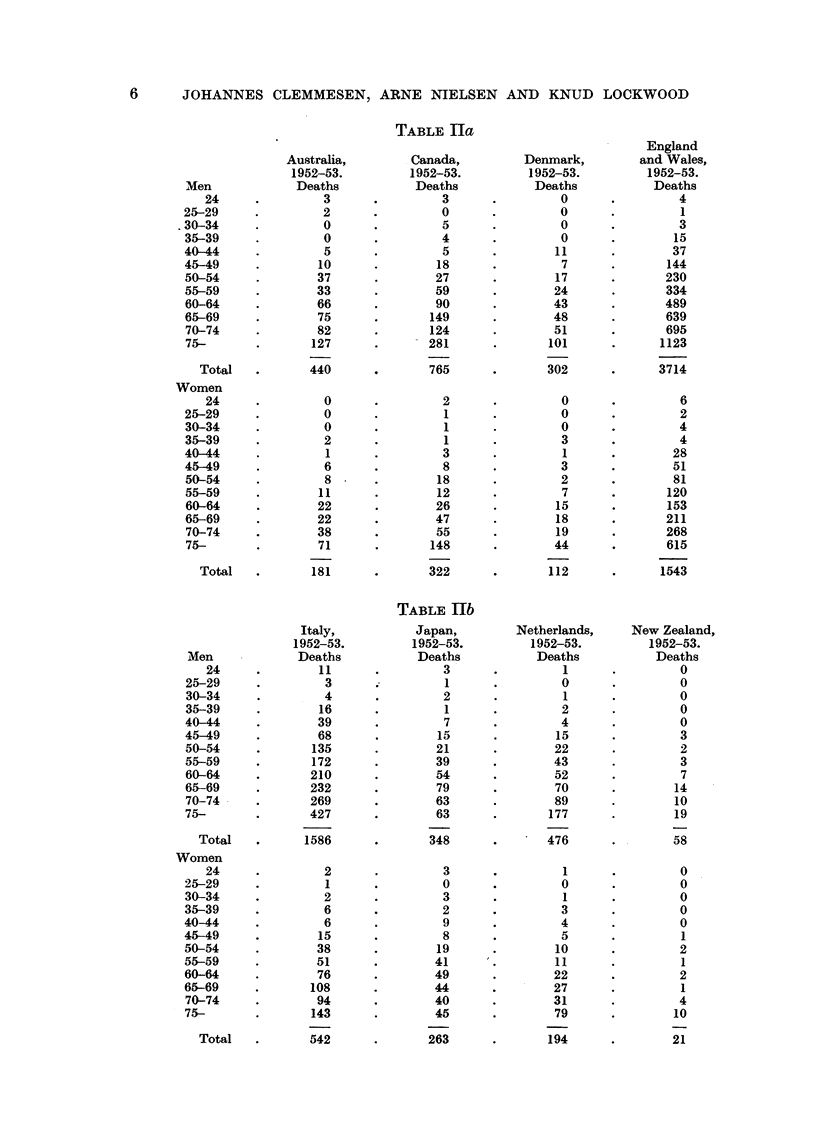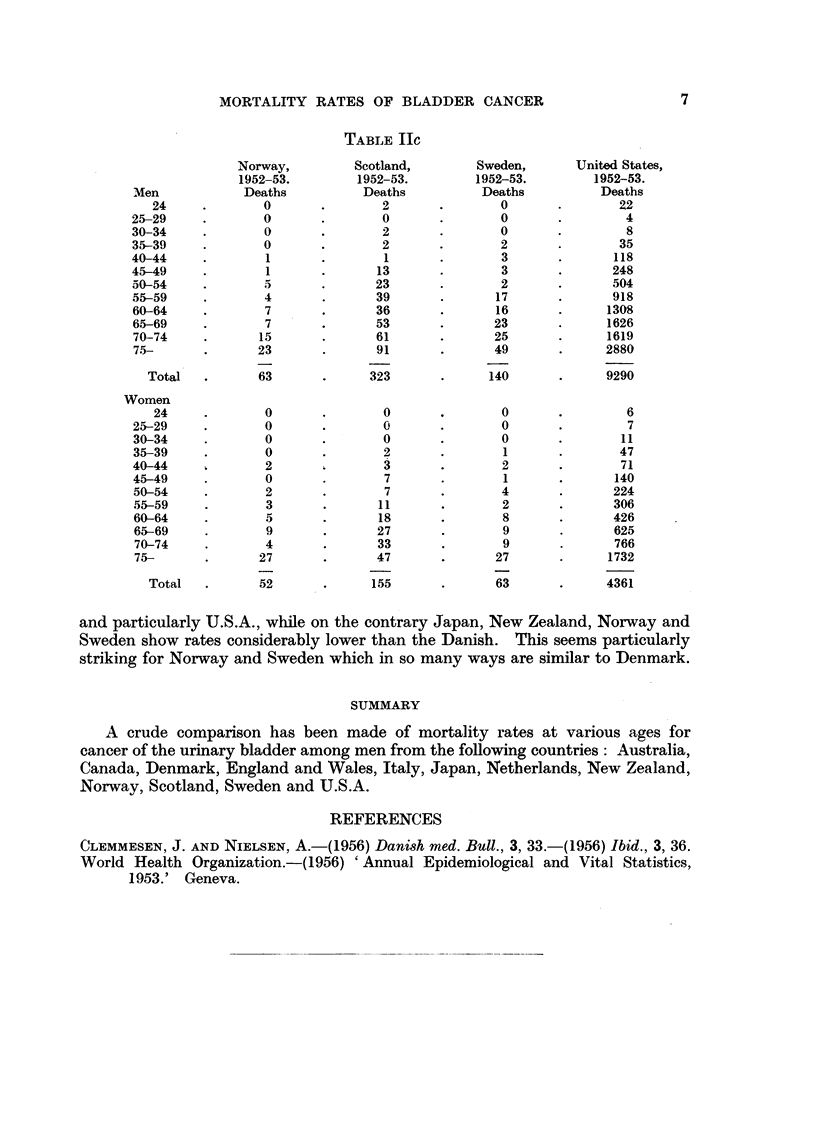# Mortality Rates for Cancer of Urinary Bladder in Various Countries

**DOI:** 10.1038/bjc.1957.1

**Published:** 1957-03

**Authors:** Johannes Clemmesen, Arne Nielsen, Knud Lockwood


BRITISH JOURNAL OF CANCER

VOL. XI MARCH, 1957 NO. 1

### MORTALITY RATES FOR CANCER OF URINARY BLADDER IN VARIOUS COUNTRIES

JOHANNES CLEMMESEN, ARNE NIELSEN AND KNUD LOCKWOOD

*From the Danish Cancer Registry under the National Anti-Cancer League, Strandboulevard 49, København Ø, Denmark*

Received for publication November 17, 1956

THE recent demonstration of an increase in incidence among men in Copenhagen of papillomata and carcinomata of the urinary bladder, reported by two of us (Clemmesen and Nielsen, 1956) and illustrated in Fig. 1, has induced us to compare as far as possible the figures for this disease from a number of countries. It is realized by the authors that a real and final comparison of such figures can only be undertaken through direct collaboration between experts from countries concerned, so that the present publication will only serve to draw the attention of such experts to the possibility that the increase observed in Copenhagen may apply also to other areas.

At present such comparison will have to be limited to mortality rates because incidence figures are only available for a few countries. Many countries do not even report specific mortality figures for tumours of the bladder, but this difficulty has been overcome through the courtesy of Dr. Y. Biraud, The Epidemiological and Statistical Division of World Health Organization, Geneva, who has kindly placed the basic figures for this study at our disposal.

A more important limitation of our study is that these figures will refer only to cancer, which exposes them to possible errors in two ways : First, papillomata will not be included, which lesions, as far as etiology studies are concerned, are probably as important as the fully developed cancers into which most of them finally develop. Probably papillomata represent a larger proportion of bladder tumours in areas where the latter are increasing in frequency than where this is not the case. Second, it is reasonable to assume that cure is more easy to attain in cases of papilloma, so that differences between countries in the proportion of papillomata as well as in the efficiency of therapy may both influence our figures.

On the other hand the Copenhagen figures suggest that an increase may be observed in cancer cases alone, and in order to give an idea of the interrelation between morbidity and mortality figures as far as Denmark is concerned, we have given the age distribution of cases as well as of deaths for all Denmark in Fig. 2 and Table I together with morbidity rates at various ages for men in Copenhagen for the two subsequent five-year periods concerned. Maybe other areas with rates similar to those for all Denmark may comprise subdivisions in which an increase in cases similar to the increase observed in Copenhagen may appear on more detailed study, thus facilitating investigations into aetiology.

Fig. 3 to 13 illustrate mortality rates for vesical cancer among men at various ages per 100,000 inhabitants. The basic figures are given in Table II, and population figures have been taken from World Health Organization's *Annual Epidemiological and Vital Statistics* (1956).

It should be mentioned that differences in age groups beyond 65 should be taken with some reservation, first because the populations in these age groups are limited so that relatively few cases may account for the differences, and second

FIG. 1.—Crude incidence rates per 100,000 for cases of cancer and of papilloma of urinary bladder, ureter and urethra among women and men in Danish capital, provincial towns and rural areas from 1943–53.

because the last point has been determined for the groups aged over 75 as a whole. We have not deemed it advisable for this orientating study to make any calculations of standard error.

FIG. 2.—Mortality and morbidity rates at various ages for tumours of urinary bladder among men in Denmark and Denmark's capital.
.......... Mortality rates for men, Denmark, 1952–53, cancer of bladder and urethra (W.H.O. Code No. 181).
———— Morbidity rates for men, Denmark, 1948–52, cancer and papillomata of bladder, ureter, urethra.
·—·—·—· Morbidity rates for men, capital, 1943–47, cancer and papillomata of bladder, ureter, urethra.
— — — — Morbidity rates for men, capital, 1948–52, cancer and papillomata of bladder, ureter, urethra.

It appears from the diagrams that the age distribution of deaths per 100,000 follows approximately the same course as the Danish curve for the following countries : Australia, Canada, England and Wales, Italy, Netherlands, Scotland,

TABLE I.—*Carcinoma and Papilloma of Urinary Bladder, Ureter and Urethra*

| | Morbidity rates per 100,000 for men at various ages | | |
|---|---|---|---|
| Age | Greater Copenhagen, 1943–47 | Greater Copenhagen, 1948–52 | All Denmark, 1948–52 |
| 24 | 0·0 | 0·C | 0·0 |
| 25–29 | 2·1 | 0·6 | 0·4 |
| 30–34 | 1·0 | 1·7 | 0·9 |
| 35–39 | 1·1 | 3·2 | 1·1 |
| 40–44 | 2·4 | 5·0 | 3·6 |
| 45–49 | 8·0 | 15·6 | 8·1 |
| 50–54 | 19·7 | 27·1 | 14·5 |
| 55–59 | 20·8 | 37·2 | 21·2 |
| 60–64 | 31·1 | 64·3 | 30·1 |
| 65–69 | 35·5 | 73·6 | 42·5 |
| 70–74 | 69·5 | 99·8 | 57·9 |
| 75– | 100·5 | 126·0 | 75·7 |

Fig. 3.

Fig. 4.

Fig. 5.

Fig. 6.

Fig. 7.

Fig. 8.

Fig. 9.

Fig. 10.

Fig. 11.

Fig. 12.

Fig. 13.

Fig. 3–13.—Mortality rates at various ages for cancer of urinary bladder and urethra (W.H.O. Code No. 181) among men in various countries. The dotted line represents the curve for Denmark. Fig. 3.—Australia, 1952–53. Fig. 4.—Canada, 1952–53. Fig. 5.—England and Wales, 1952–53. Fig. 6.—Italy, 1952–53. Fig. 7.—Netherlands, 1952–53. Fig. 8.—Scotland, 1952–53. Fig. 9.—U.S.A., 1952–53. Fig. 10.—Japan, 1952–53. Fig. 11.—New Zealand, 1952–53. Fig. 12.—Norway, 1952–53. Fig. 13.—Sweden, 1952–53.

TABLE IIa

| | Australia, 1952–53. Deaths | Canada, 1952–53. Deaths | Denmark, 1952–53. Deaths | England and Wales, 1952–53. Deaths |
|---|---|---|---|---|
| Men | | | | |
| 24 | 3 | 3 | 0 | 4 |
| 25–29 | 2 | 0 | 0 | 1 |
| 30–34 | 0 | 5 | 0 | 3 |
| 35–39 | 0 | 4 | 0 | 15 |
| 40–44 | 5 | 5 | 11 | 37 |
| 45–49 | 10 | 18 | 7 | 144 |
| 50–54 | 37 | 27 | 17 | 230 |
| 55–59 | 33 | 59 | 24 | 334 |
| 60–64 | 66 | 90 | 43 | 489 |
| 65–69 | 75 | 149 | 48 | 639 |
| 70–74 | 82 | 124 | 51 | 695 |
| 75– | 127 | 281 | 101 | 1123 |
| Total | 440 | 765 | 302 | 3714 |
| Women | | | | |
| 24 | 0 | 2 | 0 | 6 |
| 25–29 | 0 | 1 | 0 | 2 |
| 30–34 | 0 | 1 | 0 | 4 |
| 35–39 | 2 | 1 | 3 | 4 |
| 40–44 | 1 | 3 | 1 | 28 |
| 45–49 | 6 | 8 | 3 | 51 |
| 50–54 | 8 | 18 | 2 | 81 |
| 55–59 | 11 | 12 | 7 | 120 |
| 60–64 | 22 | 26 | 15 | 153 |
| 65–69 | 22 | 47 | 18 | 211 |
| 70–74 | 38 | 55 | 19 | 268 |
| 75– | 71 | 148 | 44 | 615 |
| Total | 181 | 322 | 112 | 1543 |

TABLE IIb

| | Italy, 1952–53. Deaths | Japan, 1952–53. Deaths | Netherlands, 1952–53. Deaths | New Zealand, 1952–53. Deaths |
|---|---|---|---|---|
| Men | | | | |
| 24 | 11 | 3 | 1 | 0 |
| 25–29 | 3 | 1 | 0 | 0 |
| 30–34 | 4 | 2 | 1 | 0 |
| 35–39 | 16 | 1 | 2 | 0 |
| 40–44 | 39 | 7 | 4 | 0 |
| 45–49 | 68 | 15 | 15 | 3 |
| 50–54 | 135 | 21 | 22 | 2 |
| 55–59 | 172 | 39 | 43 | 3 |
| 60–64 | 210 | 54 | 52 | 7 |
| 65–69 | 232 | 79 | 70 | 14 |
| 70–74 | 269 | 63 | 89 | 10 |
| 75– | 427 | 63 | 177 | 19 |
| Total | 1586 | 348 | 476 | 58 |
| Women | | | | |
| 24 | 2 | 3 | 1 | 0 |
| 25–29 | 1 | 0 | 0 | 0 |
| 30–34 | 2 | 3 | 1 | 0 |
| 35–39 | 6 | 2 | 3 | 0 |
| 40–44 | 6 | 9 | 4 | 0 |
| 45–49 | 15 | 8 | 5 | 1 |
| 50–54 | 38 | 19 | 10 | 2 |
| 55–59 | 51 | 41 | 11 | 1 |
| 60–64 | 76 | 49 | 22 | 2 |
| 65–69 | 108 | 44 | 27 | 1 |
| 70–74 | 94 | 40 | 31 | 4 |
| 75– | 143 | 45 | 79 | 10 |
| Total | 542 | 263 | 194 | 21 |

TABLE IIc

| Men | Norway, 1952–53. Deaths | Scotland, 1952–53. Deaths | Sweden, 1952–53. Deaths | United States, 1952–53. Deaths |
|---|---|---|---|---|
| 24 | 0 | 2 | 0 | 22 |
| 25–29 | 0 | 0 | 0 | 4 |
| 30–34 | 0 | 2 | 0 | 8 |
| 35–39 | 0 | 2 | 2 | 35 |
| 40–44 | 1 | 1 | 3 | 118 |
| 45–49 | 1 | 13 | 3 | 248 |
| 50–54 | 5 | 23 | 2 | 504 |
| 55–59 | 4 | 39 | 17 | 918 |
| 60–64 | 7 | 36 | 16 | 1308 |
| 65–69 | 7 | 53 | 23 | 1626 |
| 70–74 | 15 | 61 | 25 | 1619 |
| 75– | 23 | 91 | 49 | 2880 |
| Total | 63 | 323 | 140 | 9290 |
| Women | | | | |
| 24 | 0 | 0 | 0 | 6 |
| 25–29 | 0 | 0 | 0 | 7 |
| 30–34 | 0 | 0 | 0 | 11 |
| 35–39 | 0 | 2 | 1 | 47 |
| 40–44 | 2 | 3 | 2 | 71 |
| 45–49 | 0 | 7 | 1 | 140 |
| 50–54 | 2 | 7 | 4 | 224 |
| 55–59 | 3 | 11 | 2 | 306 |
| 60–64 | 5 | 18 | 8 | 426 |
| 65–69 | 9 | 27 | 9 | 625 |
| 70–74 | 4 | 33 | 9 | 766 |
| 75– | 27 | 47 | 27 | 1732 |
| Total | 52 | 155 | 63 | 4361 |

and particularly U.S.A., while on the contrary Japan, New Zealand, Norway and Sweden show rates considerably lower than the Danish. This seems particularly striking for Norway and Sweden which in so many ways are similar to Denmark.

### SUMMARY

A crude comparison has been made of mortality rates at various ages for cancer of the urinary bladder among men from the following countries : Australia, Canada, Denmark, England and Wales, Italy, Japan, Netherlands, New Zealand, Norway, Scotland, Sweden and U.S.A.

### REFERENCES

CLEMMESEN, J. AND NIELSEN, A.—(1956) *Danish med. Bull.*, **3**, 33.—(1956) *Ibid.*, **3**, 36.

World Health Organization.—(1956) 'Annual Epidemiological and Vital Statistics, 1953.' Geneva.